# Negative correlation between maorophage proooagulant and migraion ability in the course of inflammation

**DOI:** 10.1155/S0962935196000397

**Published:** 1996-09

**Authors:** I. Vilić, L. M. Prokić

**Affiliations:** 1 Institute of Physiology Medical Faculty Belgrade University PO Box 783 Belgrade 11001 Serbia

## Abstract

The importance of macrophage procoagulant activity (PCA) to cell migration is presumed. In this study we assayed the relationship between the two functions in guinea-pig peritoneal resident macrophages and cells elicited by a sterile inflammation induction, which lasted up to 6 days. The findings pointed to an *in vivo* induction of PCA in macrophages, which declined with time during inflammation. A clear negative correlation between PCA and random migration ability was demonstrated. Our results suggest that the local induction of coagulation by macrophages may immobilize the cells at the site of inflammation.


Mediators of Inflammation 5, 271–275 (1996)

### Negative correlation between macrophage procoagulant and migration ability in the course of inflammation

I. Vilić[CA] and L. M. Prokić

Institute of Physiology, Medical Faculty, Belgrade University, PO Box 783, 11001 Belgrade, Yugoslavia

[CA]Corresponding Author

**The importance of macrophage procoagulant activity (PCA) to cell migration is presumed. In this study we assayed the relationship between the two functions in guinea-pig peritoneal resident macrophages and cells elicited by a sterile inflammation induction, which lasted up to 6 days. The findings pointed to an *in vivo* induction of PCA in macrophages, which declined with time during inflammation. A clear negative correlation between PCA and random migration ability was demonstrated. Our results suggest that the local induction of coagulation by macrophages may immobilize the cells at the site of inflammation.**

**Key words:** Inflammation, Macrophage, Procoagulant activity, Random migration

### Introduction

Among the rich macrophage secretory repertoire, their procoagulant activity (PCA) is one of the most interesting.[1,2] Mononuclear phagocyte cells may produce various coagulation factors thus expressing a different secretory phenotype. It is also demonstrated that monocytes/macrophages possess fibrinogen receptors,[3] as well as fibrinogen/fibrin linked in a granular or net-like form.[4] The *in vivo* significance of macrophage PCA is still obscure. It is presumed that PCA contributes to the pathogenesis of some autoimmune and other immunological diseases, to graft rejection and to delayed hypersensitivity.[5–11] Besides, mononuclear phagocyte PCA is presumed to be a simple reproducible correlate of delayed hypersensitivity in man and experimental animals.[12,13]

It is also implied that the induction of extravascular coagulation by macrophages causes induration at the inflammatory *locus*.[14] Thus fibrin deposition on one side probably limits the inflammatory lesion, and on the other side may arrest inflammatory cells. Therefore, the significance of monocyte/macrophage PCA for their own migration ability is supposed.[15] The previous findings were mainly concerned with the locomotion of activated macrophages in delayed hypersensitivity reactions. To our knowledge, the development of macrophage PCA during inflammation, especially in relation to macrophage motility, has never been examined.

In this paper we tested the development of PCA in guinea-pig peritoneal macrophages during sterile inflammation, as well as the relationship between PCA and those cells' random migration. Our results point to PCA induction at the inflammatory site and its negative correlation with macrophage migration potency.

### Materials and Methods

*Animals:* Outbred guinea-pigs of both sexes, weighing 350–450 g, 5–7 per group, were used. All experiments were performed two to three times to ensure that the results were consistent and the representative experiments were shown.

*Peritoneal macrophages:* To obtain macrophages, peritoneal cavities were washed with Hanks's balanced salt solution (HBSS) supplemented with 0.5 IU/ml of heparin ('Galenika', Belgrade, Yugoslavia). Resident macrophages were harvested by the lavage of cavities of untreated animals, and elicited ones at days 3 (3-day macrophages) and 6 (6-day macrophages) after induction of a sterile peritoneal inflammation with 10 ml of paraffin oil. According to neutral red staining[16] 67–90% cells were macrophages. The remainder of the cells consisted mainly of lymphocytes and 1–2% neutrophils. Cell viability was assessed by trypan blue exclusion and was greater than 90%.

*Preparation of serum and plasma:* Blood was prepared from normal, untreated guinea-pigs by cardiac puncture. Blood samples were clotted 20 min at room temperature, incubated overnight at 4°C, centrifuged, and pooled to obtain guinea-pig serum. Nine volumes of blood were mixed with

© 1996 Rapid Science Publishers one volume of 3.8% sodium-citrate, centrifuged for 18 min at 2 000 rpm to obtain plasma, and then 15 min at 3 000 rpm (at 4°C) to eliminate platelets. The plasma samples were pooled and stored at −70°C for up to 1 month until use. In some experiments heat-inactivated plasma was used (1 h at 56°C).

*Procoagulant activity:* The ability of macrophages to shorten the coagulation time of recalcified guinea-pig plasma was assayed either with freshly isolated cells, or after incubation (5 × $10^6$/ml) for 4–20 h in M 199 (Torlak, Belgrade, Yugoslavia) at 37°C. Introductory experiments showed that the presence of serum was not necessary for the development of macrophage PCA during cell culture. After washing, macrophages were resuspended in M 199. PCA was measured by a modified one-stage method[13] in an automatic coagulometar (Fibrintimer, Behringwerke AG, Marburg, Germany). Briefly, duplicate samples of macrophage suspensions ($10^6$ cells/ 0.1 ml M 199) were mixed with 0.1 ml plasma, warmed at 37°C, and the recalcified time was measured after the addition of 0.1 ml of 0.025 M $CaCl_2$. PCA is expressed in seconds and/or milliunits (mU) of activity per $10^6$ cells. A standard curve of the plasma recalcified time was made with decreasing logarithmic dilutions of rabbit brain thromboplastin (Blood Transfusion Institute, Belgrade, Yugoslavia; 20–0.1 μg/0.1 ml saline), added to 0.1 ml plasma. Six mg of thromboplastin was nominated as 1 000 mU of PCA. The standard thromboplastin curve was compared with that obtained by using dilutions of macrophage suspensions. The slopes of the plot of clotting time versus rabbit thromboplastin dilutions were correlated.

*Random migration testing:* The macrophage random migration was assayed by the capillary tube method.[17] Resident or elicited peritoneal exudate cells (PEC) were washed in HBSS without heparin. Erythrocytes were eliminated by lysis with 0.83% $NH_4Cl$.[18] The cells were then resuspended in M 199 (40 × $10^6$ cells/ml). Migration chambers, containing two capillaries with a cell pellet, were immediately filled with medium supplemented with 10% of guinea-pig serum, or plasma, or heat-inactivated plasma. After incubation from 4 to 44 h at 37°C, the cell migration was determined by weighing the paper cut-outs of the area of the projected image. The average migration areas of four replicates (in mg) were used for calculation of the migration index (MI): MI = test area/control area × 100.

It was found that MI values under 80, i.e. 60 or 40, represent statistically significant migration inhibition ($p < 0.05$, $p < 0.01$, $p < 0.001$, respectively). The cells were also viewed by light microscopy ( × 400; Amplival microscope, Carl Zeiss, Jena, Germany), and photographs were taken with Kodak 35 mm film. Migration chambers were opened at the end of an incubation period, and the quality of medium was examined (liquid/coagulated).

*Macrophage preincubation with thrombin:* After collection, 6-day macrophages (5 × $10^6$ cells/ml) were pre-incubated 30 min in M 199 with 1–10 U/ml of thrombin (bovine; Sigma, St Louis, MO, USA) at 37°C. The cells were then washed three times in M 199 and their random migration was tested.

*Maintenance of LPS-free conditions:* All tissue culture materials, if not of a sterile plastic disposable nature, were washed and heated to 180°C for 3 h to destroy any contaminating lipopolysaccharide (LPS). Polymyxin B sulphate (100 U/ml; Sigma, St Louis, MO, USA), an inactivator of endotoxin, was added to the culture media. The substance had no effect on both random migration and PCA.

*Statistics:* The statistical analysis was performed using the two-tail Student's *t*-test and Spearman's correlation test.

### Results

*Macrophage random migration in plasma:* To investigate whether oil-elicited macrophages possess PCA of any importance to their random migration ability, the migration was at first estimated in the presence of 10% guinea-pig plasma. Plasma strongly inhibited motility of 6-day macrophages, compared with 10% serum (Fig. 1; $p < 0.001$). The analysis of migration kinetics (the increase of migration areas in time) showed that the migration areas did not increase in the presence of plasma, in contrast to the characteristic successive enlargement in the presence of serum (results not presented). The migration inhibitory factor(s) was thermolabile, since heat-inactivated plasma did not reduce migration, and was equivalent to serum (results not presented). After the opening of migration chambers, at the end of an incubation period, a complete or partial coagulation of culture medium supplemented with plasma was observed. In the control chambers containing either plasma without cells or serum plus cells, coagulation of medium was not seen. Light microscopy and photos of macrophages from migration areas showed that they were pre-

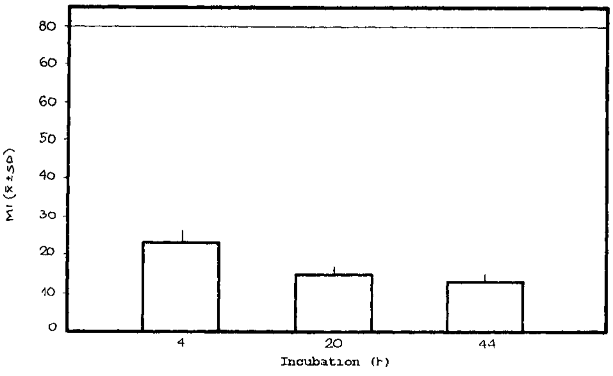

FIG. 1. The influence of 10% guinea-pig plasma *in vitro* on the random migration of oil-elicited guinea-pig peritoneal 6-day macrophages. Migration areas obtained with 10% serum are taken as controls. All experiments were performed two to three times to ensure that the results were consistent and the representative experiment is shown ($n$ = 5–7 per group). Significant inhibition, $p < 0.001$.

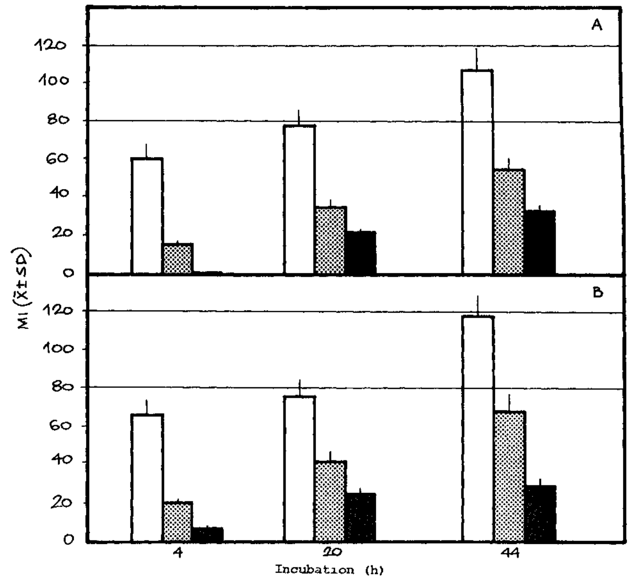

FIG. 2. The effect of macrophage preincubation with thrombin on their random migration in M 199 either (A) not or (B) supplemented with 10% serum. The 6-day macrophages were preincubated 30 min with thrombin (□ 1 U/ml, ▒ 5 U/ml, ■ 10 U/ml, in M 199). $n$ = 5–7 per group. For the significance levels see Material and Methods.

dominantly rounded in the presence of serum, whereas in plasma they displayed predominantly an elongated morphology (results not shown). The appearance of 'fibrils' in which the cellular elements appeared to be 'trapped' was also seen in migration areas from chambers with plasma.

*Effect of thrombin on macrophage random migration:* To further demonstrate that PCA influences macrophage random migration potency, the 6-day macrophages were preincubated for 30 min with 1–10 U/ml of thrombin. Controls were preincubated in M 199 with or without 10% serum. Pre-treatment with thrombin caused a strong dose-dependent migration reduction (Fig. 2). At the highest dose, 90–100% inhibition of cell locomotion was measured at 4 h. The inhibition persisted throughout the cultivation period, except with the lowest dose, but gradually weakened with time.

*PCA of peritoneal macrophages:* All types of peritoneal macrophages, i.e. resident and those harvested at days 3 and 6 of sterile inflammation, had a low basal PCA immediately after the cell collection (Fig. 3). However, the PCA was greater in 3-day macrophages compared with 6-day macrophages or resident ones. This pointed at an *in vivo* induction of PCA during the inflammatory process. *In vitro* cultivation of macrophages enhanced their PCA over basal level, to its maximum at 4–8 h. The *in vitro* PCA stimulation was more pronounced in resident and 3-day macrophages (319 ± 12 and 320 ± 30 mU/$10^6$ cells), than in 6-day macrophages (147 ± 9 mU/$10^6$ cells). Thereafter PCA returned to the initial level at 20 h, except in resident cells in which a 25% stimulation persisted at the time ($p < 0.05$). The estimation of PCA kinetics (the change in time when 0 h level was set to control one) showed that resident macrophages had the greatest potency to PCA induction (6- versus 2.5-fold in both 3-day and 6-day macrophages; results not presented).

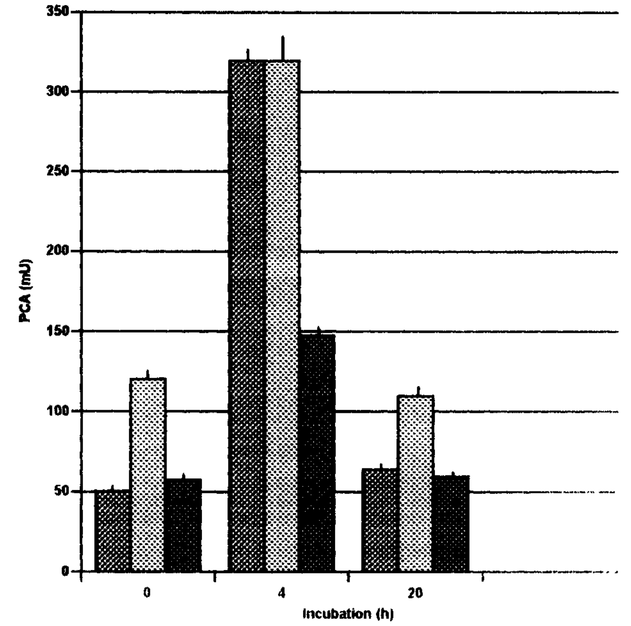

FIG. 3. The development of PCA (mU/$10^6$ cells) in resident (▓), 3-day (▒), and 6-day macrophages (■), cultivated in M 199. Data represent the means ± SD of the representative experiment ($n$ = 5–7 per group). Two to three separate experiments were done. Significant stimulation ($p < 0.001$): at 0 h, 3-day versus resident and 6-day macrophages; at 4 h, resident and 3-day versus 6-day macrophages; at 20 h, 3-day versus resident and 6-day macrophages.

**Table 1.** The random migration of guinea-pig resident and elicited macrophages. Migration areas (mean ± SD)

| Macrophages | Serum (10%) | Incubation | | |
|---|---|---|---|---|
| | | 4 h | 20 h | 44 h |
| Resident | – | 8.51 ± 0.98[a] | 10.65 ± 1.04[b] | 7.39 ± 0.82[c] |
| | + | 10.10 ± 1.07 | 20.25 ± 2.25[a] | 18.55 ± 1.94[b] |
| 3-day macrophages | – | 3.65 ± 0.25[c] | 6.97 ± 0.81[c] | 7.35 ± 0.85[c] |
| | + | 4.18 ± 0.56[c] | 9.90 ± 1.07[c] | 10.96 ± 1.36[c] |
| 6-day macrophages | – | 11.40 ± 1.28 | 20.48 ± 2.21 | 19.85 ± 2.05 |
| | + | 12.30 ± 1.28 | 26.75 ± 2.66 | 35.35 ± 3.61 |

Significant migration inhibition: [a]($p < 0.05$), [b]($p < 0.01$), [c]($p < 0.001$) when compared with 6-day macrophage migration areas.

*Random migration of peritoneal macrophages during inflammation:* The random migration was tested in parallel with PCA in the three types of macrophages (Table 1). Resident cells and mononuclear phagocytes from the earlier phase of inflammation had a lower migratory ability when compared with 6-day macrophages. It was observed on both resident and inflammatory cells, as we reported for 6-day macrophages only,[19,20] that random motility increased with time. Serum exerted its known positive chemokinetic influence,[21] as well as prolonged cell survival up to 44 h.

The subjection of the findings for PCA and random migration to the correlation test demonstrated at 4 and 20 h a significant negative relationship between the PCA and random migration capacity in all the three types of macrophages ($p < 0.05$ to $p < 0.001$).

### Discussion

It was reported that PCA induction in activated macrophages significantly reduced their locomotion *in vitro*.[10] In that paper such an influence was not attributed to elicited macrophages, in spite of evidence that they may also secrete some coagulation factors.[2] The testing of 6-day macrophage random migration in the presence of plasma seemed to us the most suitable and direct way to start the examination of the PCA–migration relationship. The results presented here show the change in shape of migrating cells, the strong reduction of the motility, as well as the coagulation of medium in such a culture condition. Because the medium coagulated only if macrophages were present in the migration chambers, it means that they started the process with their procoagulant products. The results are in line with findings that 5–50% of plasma in culture reduced macrophage motility for 27–100%.[22]

The preincubation of elicited 6-day macrophages with thrombin demonstrated the significance of PCA to motility more directly, as it caused the powerful dose-dependent migration inhibition. Thus, with 5–10 U/ml of the agent, a 80–100% locomotion reduction appeared at 4 h. The substrate to thrombin action was presumably fibrinogen bound to macrophage surface. Indeed, it has been demonstrated that about 37–68% of the elicited peritoneal macrophages possess membrane-bound fibrinogen/fibrin of plasma origin.[4] To our knowledge, the inhibitory effects of thrombin have been demonstrated only on activated guinea-pig macrophage chemotaxis, being estimated in a short period of time—usual for directed motility testing.[12] Contrary to that, in our model the influence of preincubation with thrombin on undirected migration was monitored up to 44 h. The gradual decline or even disappearance of the effect was presumably the consequences of the macrophage neutral proteases action.[12,19,23] Recently, it was reported that thrombin enhances monocyte adherence to endothelial cells *in vivo*.[24]

Although PCA induction may be assumed through some indirect data, the examinations such as ours concern both the change of PCA in macrophages during inflammation and its significance for their own migration potency, even though this is rarely present. Thus, it is supposed that the macrophage disappearing reaction in delayed hypersensitivity is mediated by PCA induction.[12] It is suggested that the loss of macrophages was due to the formation of clumps and adherence to the peritoneal mesothelium lining. The cells appearing in the peritoneal cavity, after some time, is attributed to the stimulation of their own opposite fibrinolytic activity. Our study with resident and inflammatory macrophages shows that all three types of macrophages had a small basal PCA immediately after being lavaged out of the peritoneum. The findings that PCA was the highest in 3-day macrophages and the lowest in resident ones, that resident cells exerted a better potency for PCA induction, and that coagulation inducibility was best in 'younger' inflammatory cells, speaks in favour of a gradual decrease of the PCA of mononuclear phagocytes in the course of inflammation. The parallel macrophage random motility testing further supported the view that PCA is important to cell mobility, as migration ability was negatively correlated with PCA. Some previous findings have also demonstrated the functional difference between macrophage populations. Farram *et al.*[25] reported that a mouse bone marrow macrophage cell line, maturating *in vitro*, expressed both higher PCA and sensitivity to macrophage procoagulant-inducing factor. The differences in responses to that factor were also demonstrated between different macrophage populations.[2] Resident, elicited and activated peritoneal murine macrophages were also found to differ in adherence and spreading,[26] and PCA.[27] Resident and *in vivo* activated macrophage migrated two-fold smaller out of capillary tubes than elicited ones.[22] A part of functional differences may originate from the observation that resident macrophages do not have membrane integrin LFA-1 but inflammatory cells do.[28] It should be kept in mind that elicited peritoneal macrophages are usually harvested after 3–4 days of induction, so that the data concerning PCA and migration are closer to our findings with 3-day macrophages than with 6-day macrophages.

The decrease of PCA in the course of both *in vivo* inflammation and *in vitro* cultivation may be the consequence of the enhanced opposite fibrinolytic activity, also attributed to mononuclear phagocytes.[19,29] Parallel tests of both functions point to the possible importance of a balance between procoagulant and fibrinolytic activities in the regulation of macrophage migration at sites of inflammation.[27,29,30]

The presented results imply that activation of PCA may retain macrophages at the site of inflammation thus enabling them to accomplish appropriate biological activities.

### References

1. Edgington TS. Recognition coupled responses of the monocyte: activation of coagulation pathways. *Nouv Rev Fr Hematol* 1983; **25:** 1–6.
2. Ryan J, Geczy C. Coagulation and the expression of cell-mediated immunity. *Immunol Cell Biol* 1987; **65:** 127–139.
3. Shainoff JR, Stearns DJ, DiBello PM, Hishikawa-Itoh Y. Characterization of a mode of specific binding of fibrin monomer through its amino-terminal domain by macrophages and macrophage cell-lines. *Thromb Haemostasis* 1990; **63:** 193–203.
4. Colvin RB, Dvorak HF. Fibrinogen/fibrin on the surface of the macrophages: detection, distribution, binding requirements, and possible role in macrophage adherence phenomena. *J Exp Med* 1975; **142:** 1377–1390.
5. Geczy CL, Roberts IM, Meyer P, Bernard CCA. Susceptibility and resistance to experimental autoimmune encephalomyelitis and neuritis in the guinea pig correlate with induction of procoagulant and anticoagulant activities. *J Immunol* 1984; **133:** 3026–3036.
6. Grgacic E, Bernard CCA. Cell-mediated immune response to copolymer I in multiple sclerosis measured by the macrophage procoagulant activity assay. *Int Immunol* 1990; **2:** 713–718.
7. Holdsworth SR, Tipping PG. Macrophage-induced glomerular fibrin deposition in experimental glomerulonephritis in the rabbit. *J Clin Invest* 1985; **76:** 1367–1374.
8. Devery JM, Geczy CL, Declarle D, Skerritt JH, Krillis SA. Macrophage procoagulant activity as an assay of cellular hypersensitivity to gluten peptides in coeliac disease. *Clin Exp Immunol* 1990; **82:** 333–337.
9. Rothberger H, Barringer M, Meredith J. Increased tissue factor activity of monocytes/macrophages isolated from canine allografts. *Blood* 1984; **63:** 623–628.
10. Hopper KE, Geczy CL, Davies WA. A mechanism of migration inhibition in delayed-type hypersensitivity reactions. I. Fibrin deposition on the surface of elicited peritoneal macrophages *in vivo*. *J Immunol* 1981; **126:** 1052–1058.
11. Imamura T, Iyama K-I, Takeya M, Kambara T, Nakamura S. Role of macrophage tissue factor in the delayed hypersensitivity reaction in monkey skin. *Cell Immunol* 1993; **152:** 614–622.
12. Geczy CL, Meyer PA. Leukocyte procoagulant activity in man: an *in vitro* correlate of delayed-type hypersensitivity. *J Immunol* 1982; **128:** 331–336.
13. Geczy CL, Farram E, Moon DK, Meyer PA, McKenzie IFC. Macrophage procoagulant activity as a measure of cell-mediated immunity in the mouse. *J Immunol* 1983; **130:** 2743–2749.
14. Malik AB, Lee BC, van der Zee H, Johnson A. The role of fibrin in the genesis of pulmonary edema after embolization in dogs. *Circ Res* 1979; **45:** 120–125.
15. Geczy CL, Hopper KE. A mechanism of migration inhibition in delayed-type hypersensitivity reactions. II. Lymphokines promote procoagulant activity of macrophages *in vitro*. *J Immunol* 1981; **126:** 1059–1065.
16. Cohn ZA, Wiener, E. The particulate hydrolases of macrophages. II. Biochemical and morphological response to particle ingestion. *J Exp Med* 1963; **118:** 1009–1019.
17. Federlin K, Maini RS, Russell AS, Dumonde DC. A micro method for peripheral leukocyte migration in tuberculin sensitivity. *J Clin Pathol* 1971; **24:** 533–540.
18. Boyle W. An extension of the $^{51}$Cr-release assay for the estimation of mouse cytotoxins. *Transplantation* 1968; **6:** 761–764.
19. Vilić IM, Prokić LJM, Spuzić IV. Modulation of guinea-pig peritoneal macrophage migration *in vitro*: effects of protease inhibitors. *Immunol Lett* 1987; **14:** 271–276.
20. Prokić LJ, Vilić I. Influence of protein synthesis inhibitors on the migration ability of peritoneal macrophages *in vitro*. *Period Biol* 1986; **88** (suppl 1): 185–186.
21. Vilić I, Prokić LJ, Spuzić I. Susceptibility of macrophage random migration and chemokinesis to methylprednisolone. *Period Biol* 1986; **88** (suppl 1): 143–144.
22. Bianco C, Gotze O, Cohn ZA. Regulation of macrophage migration by products of complement system. *Proc Natl Acad Sci USA* 1979; **76:** 888–891.
23. Vilić I, Prokić LJ. Modulation of guinea-pig peritoneal macrophage migration *in vitro*: effects of acid-treated horse serum and Urbason. *Period Biol* 1986; **1:** 3–8.
24. Shanker R, de la Motte CA, DiCorleto PE. Thrombin stimulates PDGF production and monocyte adhesion through distinct intracellular pathways in human endothelial cells. *Am J Physiol* 1992; **262** (Cell Physiol 31): C199–C206.
25. Farram E, Geczy CL, Moon DK, Hopper K. The ability of lymphokines and lipopolysaccharide to induce procoagulant activity in mouse macrophage cell line. *J Immunol* 1983; **130:** 2750–2755.
26. Polliack A, Gordon S. Scanning electron microscopy of murine macrophages. Surface characteristics during maturation, activation and phagocytosis. *Lab Invest* 1975; **33:** 469–467.
27. Karhumaki E, Saravo L, Helin H. Cells of the human myelomonocytic line RC-2A synthesize tissue factor-like procoagulant and urokinase-type plasminogen activator. *J Leuk Biol* 1987; **42:** 36–42.
28. Anderson DC, Springer TA. Leukocyte adhesion deficiency: an inherited defect in the Mac-1, LFA-1 and p150,95 glycoproteins. *Ann Rev Med* 1987; **381:** 175–194.
29. Chapman HA Jr, Vavrin Z, Hibbs JB Jr. Coordinate expression of macrophage procoagulant and fibrinolytic activity *in vitro* and *in vivo*. *J Immunol* 1983; **130:** 261–266.
30. Perez RL, Roman, Staton GW Jr, Hunter RL. Extravascular coagulation and fibrinolysis in murine lung inflammation induced by the mycobacterial cord factor trehalose-6,6′-dimicolate. *Am J Respir Crit Care Med* 1994; **149:** 510–518.

ACKNOWLEDGEMENT. This work was supported by a grant of Republic Serbia Research Fund.

**Received 23 May 1996; accepted 11 June 1996**
